# Penetration of HIV-1 Tat_47–57_ into PC/PE Bilayers Assessed by MD Simulation and X-ray Scattering

**DOI:** 10.3390/membranes5030473

**Published:** 2015-09-22

**Authors:** Chris Neale, Kun Huang, Angel E. García, Stephanie Tristram-Nagle

**Affiliations:** 1 Department of Physics, Applied Physics and Astronomy, Rensselaer Polytechnic Institute, 110 8th St, Troy, NY 12180-3590, USA; E-Mails: candrewn@gmail.com (C.N.); harryhuangkun@gmail.com (K.H.); angel@rpi.edu (A.E.G.); 2 Center for Biotechnology and Interdisciplinary Studies, Rensselaer Polytechnic Institute, 110 8th St, Troy, NY 12180-3590, USA; 3 Biological Physics Group, Department of Physics, Carnegie Mellon University, 5000 Forbes Avenue, Pittsburgh, PA 15213, USA

**Keywords:** cell-penetrating peptide, lipid bilayers, peptide translocation, molecular dynamics simulation, X-ray scattering, HIV-1 Tat

## Abstract

The interactions of the basic, cell-penetrating region (Y_47_GRKKRRQRRR_57_) of the HIV-1 Tat protein with dioleoylphosphatidylcholine (DOPC) bilayers were previously assessed by comparing experimental X-ray diffuse scattering with atomistic molecular dynamics simulations. Here, we extend this investigation by evaluating the influence of phosphatidylethanolamine (PE) lipids. Using experimental bilayer form factors derivedfrom X-ray diffuse scattering data as a guide, our simulations indicate that Tat peptides localize close to the carbonyl-glycerol group in the headgroup region of bilayers composed of either DOPC or DOPC:DOPE (1:1) lipid. Our results also suggest that Tat peptides may more frequently insert into the hydrophobic core of bilayers composed of PC:PE (1:1) lipids than into bilayers composed entirely of PC lipids. PE lipids may facilitate peptide translocation across a lipid bilayer by stabilizing intermediate states in which hydrated peptides span the bilayer.

## 1. Introduction

The HIV-1 viral genome encodes a protein called Tat that enhances viral transcription [1]. A peptide derived from this protein (Y_47_GRKKRRQRRR_57_), hereafter referred to as Tat_47–57_, is capable of penetrating cell membranes [2]. Many cell penetrating peptides (CPPs), including those derived from Tat, are capable of carrying cargo into live cells [3,4]. It is controversial, however, if endocytosis is involved, which would require ATP [5,6,7,8,9,10]. To address this question, many biophysical studies have used simplified models of biological membranes composed of a small number of lipid types including phosphatidylcholine (PC), phosphatidylethanolamine (PE), phosphatidylserine (PS), and phosphatidylglycerol (PG). For example, Mishra *et al.* reported that rhodamine-tagged Tat_47–57_ enters giant unilamellar vesicles (GUVs) composed of PS/PC (1:4 mole ratio) immeasurably slowly, but crosses a GUV composed of PS/PC/PE (1:2:1) lipids within 30 s [11]. In another experiment, fluorescently labeled Tat_57–48_ did not enter GUVs containing only PC with 20 mole % cholesterol, but translocated rapidly when PS or PE was included with PC [12]. Therefore, Tat-derived peptides can translocate across bilayers without ATP, but this depends on the lipid type. The mechanism by which bilayer translocation occurs remains unknown.

Previously, X-ray scattering was used to characterize Tat_47–57_-induced perturbation of simplified membrane mimetics with varying compositions of PC, PE, PS, phosphatidylinositol (PI) lipids, and cholesterol [13]. Concurrently, molecular dynamics (MD) simulations were used to generate higher-resolution models of Tat_47–57_ interacting with PC bilayers, which were scored based on their goodness of fit to experimental values [13]. Since simulations of solute immersion in a lipid bilayer are prone to systematic sampling errors on achievable simulation timescales [14,15], we employed a modelization protocol in which both area/lipid (A_L_) and Tat_47–57_ immersion depth were restrained to a series of prescribed values. The advantages of this approach are that it does not require brute-force equilibration in these two slowly-relaxing degrees of freedom (A_L_ and peptide immersion depth), and that it does not rely entirely upon the force field parameters to generate accurate models [16,17]. These previous experimental and computational results indicated that the center of mass of Tat_47–57_ most likely resides in the PC bilayer’s headgroup region, ~18 Å from the bilayer center [13].

Here, the simulation approach employed in the previous work is extended to include mixed PC:PE (1:1) bilayers. We identify simulation conditions whose resulting form factors are maximally consistent with experimental X-ray scattering form factor data and then use these simulations to obtain plausible atomistic depictions of Tat_47–57_-bilayer interactions. Interestingly, while the experimental X-ray scattering form factors of Tat_47–57_ in PC:PE (1:1) bilayers are most consistent with simulation models in which the center of mass of Tat_47–57_ resides in the bilayer's headgroup region, we also obtain good fits to experiment for some simulations in which Tat_47–57_ is much more deeply inserted, ~8 Å from the bilayer center.

## 2. Results

Previously, X-ray diffuse scattering were collected for DOPC, DOPC:DOPE (3:1), DOPC:DOPE (1:1), DOPC:DOPS (3:1) and a nuclear membrane mimic [13]. The Scattering Density Profile (SDP) program [18], which is a self-contained modeling program, indicated that either a headgroup position or an internal, hydrocarbon position was equally favorable for Tat_47–57_ in several lipid mixtures. This bimodal result was the motivation to compare the form factors from experiment to those from MD simulation in which Tat_47–57_ was restrained in various regions of the bilayer. In the previous work, only DOPC bilayers were compared to MD simulations, with the result that good agreement between X-ray experiment and MD simulation was found only when Tat_47–57_ was located in the headgroup region, and not in the hydrocarbon region, of the bilayers [13]. In the present work, we evaluate whether simulations can reproduce the bilayer form factor derived from the experimental X-ray data of DOPC:DOPE (1:1) mixtures by directly comparing the form factor resulting from the Fourier transform of the simulated electron density profiles to the model-free X-ray experimental form factor.

### 2.1. Neat Bilayers

In the absence of an applied surface tension or fixed dimensions of the unit cell, simulations of neat bilayers can reproduce experimental values of bilayer thickness and A_L_, and can provide relatively accurate predictions of lipid order parameters and X-ray form factors [19,20,21]. However, our primary objective involves the simulation of peptide-bilayer systems, for which we fix the bilayer’s A_L_ in an attempt to circumvent systematic sampling errors related to slow conformational relaxation in atomistic simulations. Therefore, our initial evaluation of a neat lipid bilayer is also conducted with systematic variation of fixed A_L_ values.

**Figure 1:**
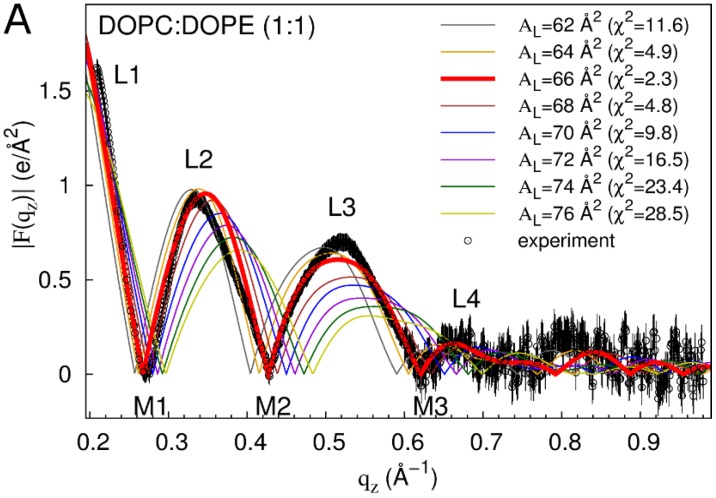

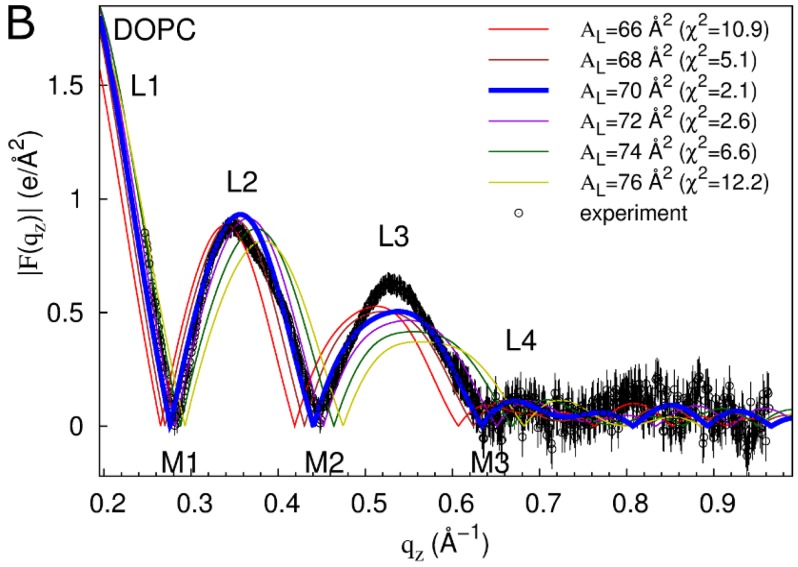
Form factors of neat bilayers from experiment and simulation. |F(q_z_)| based on out-of-plane scattering intensities from experiment are compared to the computed |F(q_z_)| from simulations at different values of A_L_ (χ^2^ values in parentheses) for DOPC:DOPE (1:1) (**A**) and DOPC (**B**). Out-of-plane scattering lobes (L) and minima (M) are numbered. Experimental data are averaged over three (**A**) and four (**B**) experiments. Vertical lines depict experimental error estimates (see Methods). Experimental data were scaled to the simulations at A_L_ = 66 Å^2^ (**A**) and A_L_ = 70 Å^2^ (**B**) (see Methods).

Of the evaluated bilayer dimensions, A_L_ values of 66 and 70 Å^2^ yield the best fit to experiment for DOPC:DOPE (1:1) and DOPC bilayers, respectively (Figure 1). At these A_L_ values, simulations reproduce the experimental form factor |F(q_z_)| quite well, capturing lobes L1–L4 and minima M1–M3 and having an overall χ^2^ measure of the goodness of fit (see Methods) between |F(q_z_)| from the simulation and experiment of 2.3 and 2.1 for DOPC:DOPE (1:1) and DOPC bilayers, respectively (Figure 1). However, the simulated |F(q_z_)| do not capture the skew of lobe 2 towards smaller values of q_z_ which is likely an experimental artifact. More importantly, the simulations slightly over-estimate the height of lobe 2 relative to lobes 1 and 3 (Figure 1). These differences also appear in comparisons of experiment and simulations conducted with variable A_L_ [19] and so are not directly related to the suppression of fluctuations in A_L_ (Figure S1).

### 2.2. Tat_47–57_ in DOPC:DOPE (1:1) Bilayers

**Figure 2:**
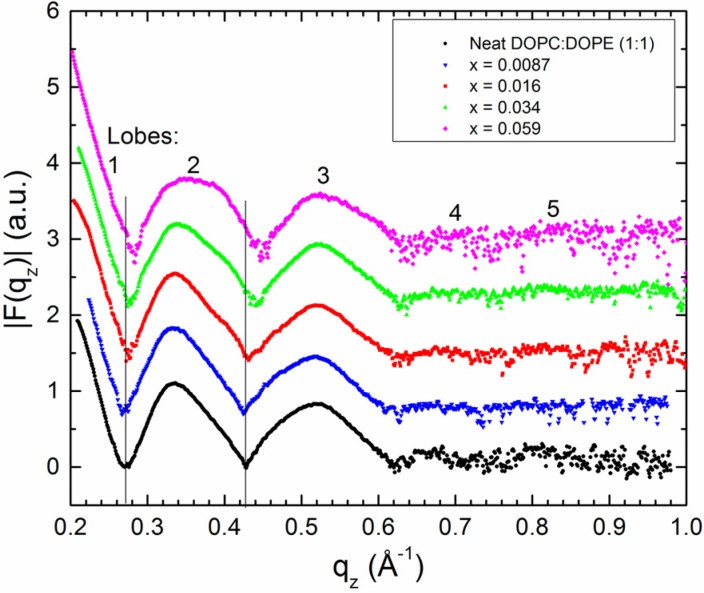
Form factors, |F(q_z_)|, from X-ray scattering data for a DOPC:DOPE (1:1) neat bilayer and increasing mole fractions, x, of Tat_47–57_. Traces are offset vertically for ease of viewing shifts in q_z_. The vertical solid lines indicate the zero positions (*i.e.*, minima) of the neat DOPC:DOPE (1:1) bilayer.

Previously, X-ray form factors were presented for a DOPC:DOPE (1:1) mixture with mole fractions of Tat_47–57_ , x = mole Tat_47–57_/(mole lipid + mole Tat_47–57_), equal to 0.0087, 0.016, 0.034, and 0.059 [13]. Form factor perturbation by Tat_47–57_ is included again here as Figure 2. At the lowest concentration of Tat_47–57_, x = 0.0087 (blue inverted triangles in Figure 2), the positions of the minima between lobes 1 and 2, and between lobes 2 and 3, shift to lower q_z_, indicating bilayer thickening. As the concentration of Tat_47–57_ increases, the experimental form factors shift to higher q_z_, indicating bilayer thinning (Figure 2).

**Figure 3:**
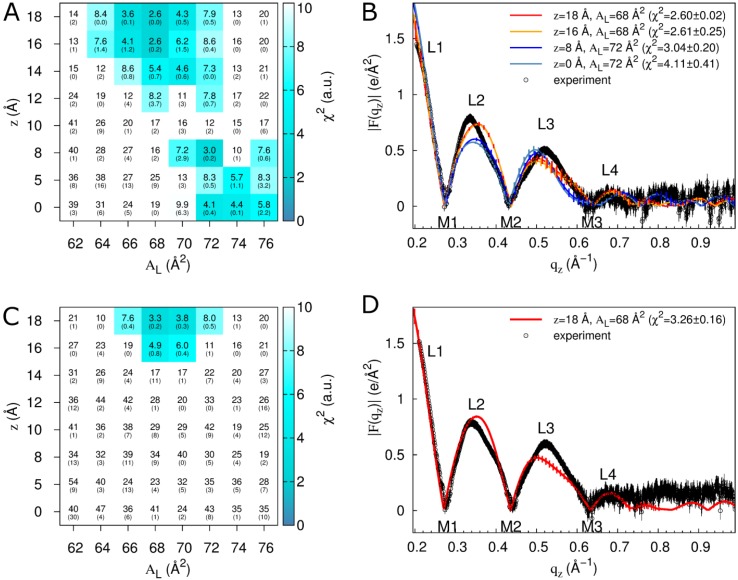
Fits of simulation to experiment for a systematic evaluation of z and A_L_ in DOPC:DOPE (1:1). Data are from simulations with two (**A**,**B**) or four (**C**,**D**) molecules of Tat_47–57_ (simulation mole fractions of 0.015 and 0.030, respectively; comparisons are to experimental data at mole fractions of 0.016 and 0.034, respectively). (**A**,**C**) Color and inset numbers represent χ^2^ values (standard deviations of averages from repeat simulations in parentheses). (**B**,**D**) Selected form factors (χ^2^ values in parentheses). Out-of-plane scattering lobes (L) and minima (M) are numbered based on experimental data, which are averaged over two (**B**) and nine (**D**) experiments. Black vertical lines depict experimental error estimates (see Methods). Vertical lines on the simulation data depict standard deviations between two starting configurations. Experimental data in (**B)** and (**D**) were scaled to the simulations at z = 18 Å, A_L_ = 68 Å^2^ (see Methods).

To generate atomistic models of the Tat_47–57_ interaction with a DOPC:DOPE (1:1) bilayer, we conducted simulations in which we systematically varied peptide distance from the bilayer center along its normal, z, and bilayer A_L_ using peptide mole fractions of either 0.015 or 0.030 (128 lipids and 2 or 4 peptides, respectively). These are the same two concentrations previously investigated with MD simulations and pure DOPC bilayers [13]. Form factors computed from these simulations are fit to the experimental data to identify those simulations that are maximally consistent with X-ray scattering results. χ^2^ values reporting the goodness of fit between simulation and experiment are shown in Figure 3A,C for simulated/experimental peptide mole fractions of 0.015/0.016 and 0.030/0.034, respectively. Comparisons of form factors from experiment and selected simulations are shown in Figure 3B,D.

**Figure 4:**
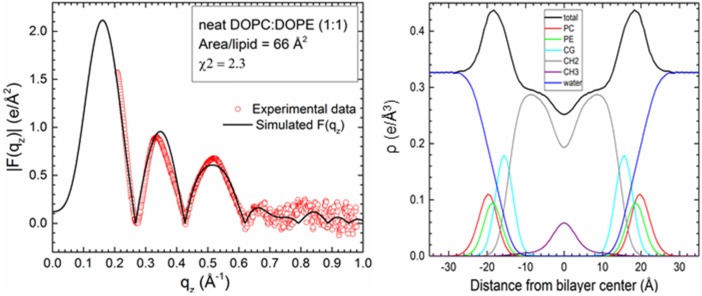
Detailed comparison of |F(q_z_)| (**left**) between neat DOPC:DOPE (1:1) experimental data points (open red circles) and simulation |F(q_z_)| (black line) at the A_L_ that yielded the smallest χ^2^ (A_L_ = 66 Å^2^, χ^2^ = 2.3). Experimental data were scaled to the simulated |F(q_z_)|. Electron density profile (**right**) from the same simulation with component groups indicated in the figure legend. PC, phosphatidylcholine and PE, phosphatidylethanolamine headgroups; CG, carbonyl-glycerol; CH2, methylenes and methines in hydrocarbon chains; CH3, terminal methyl group.

**Figure 5:**
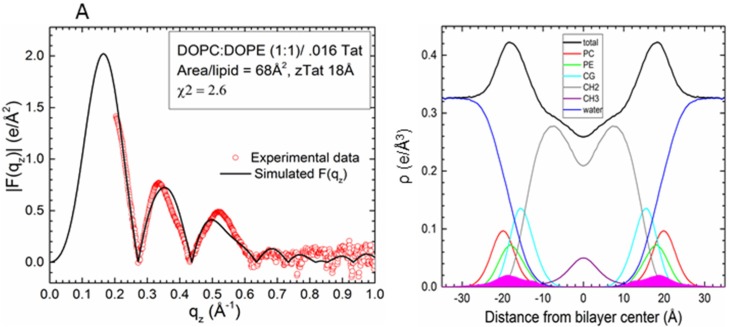

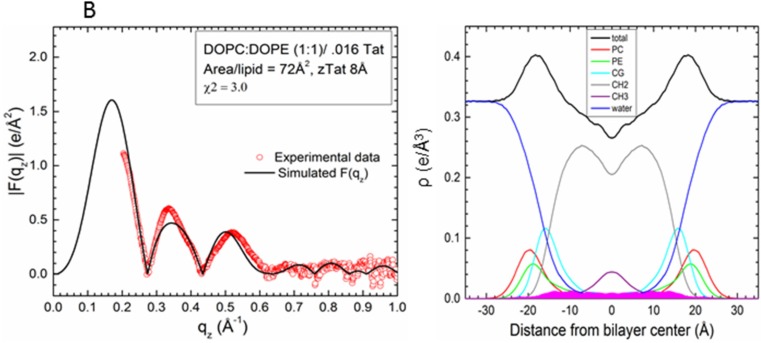
Detailed comparison of |F(q_z_)| (**left**) between DOPC:DOPE (1:1) with a Tat_47–57_ mole fraction of 0.015/0.016 (simulation/experiment) at the values of z and A_L_ that yielded the smallest χ^2^ values: (**A**) z = 18 Å, A_L_ = 68 Å^2^, χ^2^ = 2.60 ± 0.02 and (**B**) z = 8 Å, A_L_ = 72 Å^2^, χ^2^ = 3.04 ± 0.20. Experimental data were scaled to the simulated |F(q_z_)|. Electron density profiles (**right**) of the same simulations with component groups indicated in the figure legend. Tat_47–57_ electron density shown in solid magenta; other abbreviations as in Figure 4.

**Figure 6:**
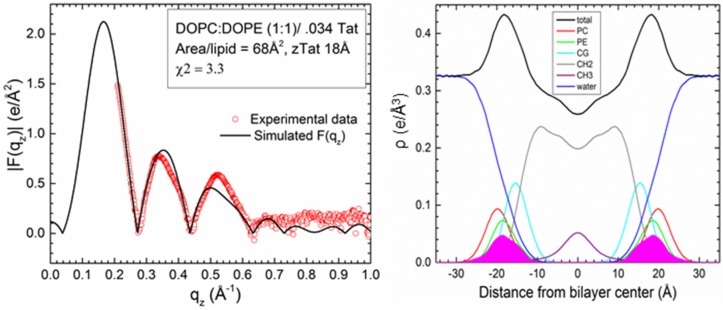
Detailed comparison of |F(q_z_)| (**left**) between DOPC:DOPE (1:1) with a Tat_47–57_ mole fraction of 0.030/0.034 (simulation/experiment) at the value of z and A_L_ that yielded the smallest χ^2^ value: z = 18 Å, A_L_ = 68 Å^2^, χ^2^ = 3.26 ± 0.16. Experimental data were scaled to the simulated |F(q_z_)|. Electron density profile (**right**) of the same simulation with component groups indicated in the figure legend. Tat_47–57_ electron density shown in solid magenta; other abbreviations as in Figure 4.

Comparison to experiment for simulations with a Tat_47–57_ mole fraction of 0.015 indicates that these peptides reside either in the DOPC:DOPE (1:1) bilayer’s headgroup region (z = 18 Å, A_L_ = 68 Å^2^, χ^2^ = 2.60 ± 0.02; or z = 16 Å, A_L_ = 68 Å^2^, χ^2^ = 2.61 ± 0.25) or undergo much deeper insertion (z = 8 Å, A_L_ = 72 Å^2^, χ^2^ = 3.04 ± 0.20, Movie SM1) (Figure 3A,B). Conversely, the simulation data for a peptide mole fraction of 0.030 indicate that Tat_47–57_ resides exclusively in the bilayer’s headgroup region (z = 18 Å, A_L_ = 68 Å^2^, χ^2^ = 3.26 ± 0.16) (Figure 3C,D). The |F(q_z_)| (left) and corresponding electron density profile (right) calculated from those parameters that yielded the smallest χ^2^ values are shown in Figure 4, Figure 5 and Figure 6. Figure 4 shows the results for the neat DOPC:DOPE (1:1) bilayers A_L_ = 66 Å^2^, while Figure 5 shows the results for a Tat_47–57_ mole fraction of 0.015 (A) z = 18 Å, A_L_ = 68 Å^2^ (B) z = 8 Å, A_L_ = 72 Å^2^, and Figure 6 shows the results for a Tat_47–57_ mole fraction of 0.030, z = 18 Å, A_L_ = 68 Å^2^. In Figure 4, Figure 5 and Figure 6, the headgroup electron density profile is shown to extend ~2 Å further out from the bilayer center for PC (red line) than PE (green line) lipids.

Structural results for the bilayers from Figure 4, Figure 5 and Figure 6 are summarized in Table 1. Increasing concentrations of Tat_47–57_ up to x = 0.034 thin the bilayer only slightly as determined from D_HH_ (head-to-head spacing, black lines in the electron density profiles). Greater thinning was observed in the hydrocarbon thickness, 2D_C_. The greatest thinning, 2.5 Å, was determined in the 2D_C_ value for DOPC/DOPE(1:1)/2 Tats (x = 0.015) when A_L_ = 72 Å^2^ with Tat_47–57_ located at 8 Å from the bilayer center. For this sample only, there is also a large increase in area (6 Å^2^) (Table 1).

**Table 1:** Summary of DOPC:DOPE (1:1) bilayer perturbation by Tat_47–57_ insertion.

Sample	2Dc (Å)	D_HH_ (Å)	A_L_(Å^2^)	zTat (Å)
DOPC/DOPE(1:1) Control(x = 0)	29.5	36.7	66	–
DOPC/DOPE(1:1)/2Tats(x = 0.015)	28.5	36.4	68	18
DOPC/DOPE(1:1)/2Tats(x = 0.015)	27.0	36.2	72	8
DOPC/DOPE(1:1)/4Tats(x = 0.030)	28.5	36.1	68	18

To further assess the extent of systematic sampling errors in our simulations, we conducted another N = 50 simulations for each of three combinations of z and A_L_ with a Tat_47–57_ mole fraction of 0.015. Specifically, we reevaluated peptide insertion into the bilayer headgroup region at z = 18 Å and A_L_ = 68 Å^2^, and deep peptide insertion at z = 0 or 8 Å and A_L_ = 72 Å^2^, using different initial conformations in each repeat simulation. Values of χ^2^ are reproducibly low when Tat_47–57_ is inserted into the bilayer’s headgroup region (χ^2^ = 2.7 ± 0.2; Table 2). However, the mean χ^2^ values obtained from 50 simulations for deep peptide insertion (7.6 ± 4.1 and 6.4 ± 2.1 for *z* = 8 and 0 Å, respectively, Table 2) are substantially larger than the values that we obtained in our first two repeat simulations (χ^2^ = 3.0 ± 0.2 and 4.1 ± 0.4 for *z* = 8 and 0 Å, respectively, Figure 1).

**Table 2:** Distribution of χ^2^ values in 50 additional simulations of selected simulation conditions in DOPC:DOPE (1:1) with a Tat_47–57_ mole fraction of 0.015.

z (Å)	A_L_ (Å^2^)	χ^2^		
		Mean ± Std. Dev.	Min.	Max.
18	68	2.7 ± 0.2	2.3	3.4
8	72	7.6 ± 4.1	2.8	18.0
0	72	6.4 ± 2.1	3.0	13.2

**Figure 7:**
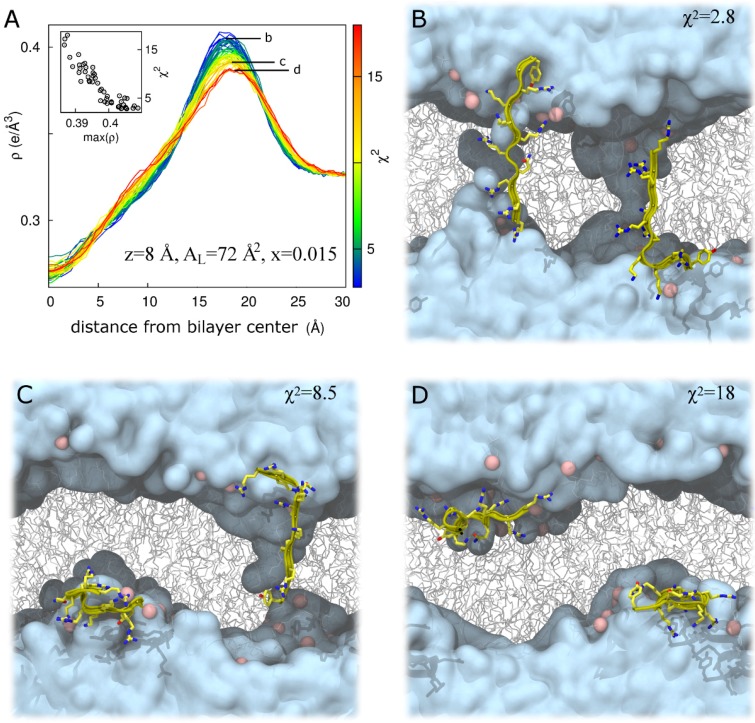
Bilayer undulations increase χ^2^ in DOPC:DOPE (1:1) at z = 8 Å, A_L_ = 72 Å^2^ for a Tat_47–57_ mole fraction of 0.015. (**A**) Total system electron density, ρ, as a function of absolute distance from the global bilayer center along its global normal for N = 52 simulations, colored by χ^2^ value representing a range from best (blue) to worst (red) fit to experiment. Inset, χ^2^ as a function of the maximum value of ρ. Labels b, c, and d in part A identify electron density curves for systems whose snapshots are shown in parts B, C, and D, respectively. (**B**–**D**) Snapshots after 100 ns of simulation in repeats with χ^2^ values of 2.8 (**B**); 8.5 (**C**); and 18 (**D**). Water is cyan, lipids are gray, lipid headgroup phosphorus atoms are pink, and protein is yellow. Protein is rendered in front of all other molecules for clarity.

The above results indicate that form factors from 100-ns simulations are relatively well converged for peptide insertion into the bilayer’s headgroup region, allowing us to identify z = 18 or 16 Å and A_L_ = 68 Å^2^ as a reliably good fit to the experimental data for a Tat_47–57_ mole fraction of 0.015 (Figure 3A,B and Table 2) and 0.030 (Figure 3C,D). Conversely, our simulations converge poorly for deeper peptide insertion (Table 2), indicating the presence of systematic sampling errors arising from the existence of multiple metastable conformations of the peptide/bilayer system whose resolution would require substantially longer simulation timescales. Of note, the electron density peak corresponding to the bilayer’s headgroup region becomes broader as χ^2^ increases for simulations at z = 8 Å, A_L_ = 72 Å^2^ (Figure 7A), but not at z = 18 Å, A_L_ = 68 Å^2^ (Figure S2). This correlation highlights artifacts that can arise when using our methodological approach. Specifically, the peptide/bilayer restraint at z = 8 Å can be satisfied in two ways. For these simulations, when χ^2^ is relatively small, the peptides generally span the bilayer, interacting with lipid headgroups from both leaflets (Figure 7B). Conversely, because the restraint relates to the global bilayer center, it can also be satisfied via bilayer undulations that position the peptide near the desired position along the global bilayer normal despite peptide residence in the lipid headgroup region and a large dissatisfaction of a local interpretation of the restraint (Figure 7C,D). The importance of controlling for bilayer undulations when comparing form factors from simulation and experiment has been noted by Braun *et al.* [22]. However, the artifact depicted in Figure 7C,D does not entirely explain the spread of χ^2^ values in simulations at z = 8 Å, A_L_ = 72 Å^2^ (Figure S3), suggesting that other slowly relaxing degrees of freedom contribute to the poor convergence of these simulations.

**Figure 8:**
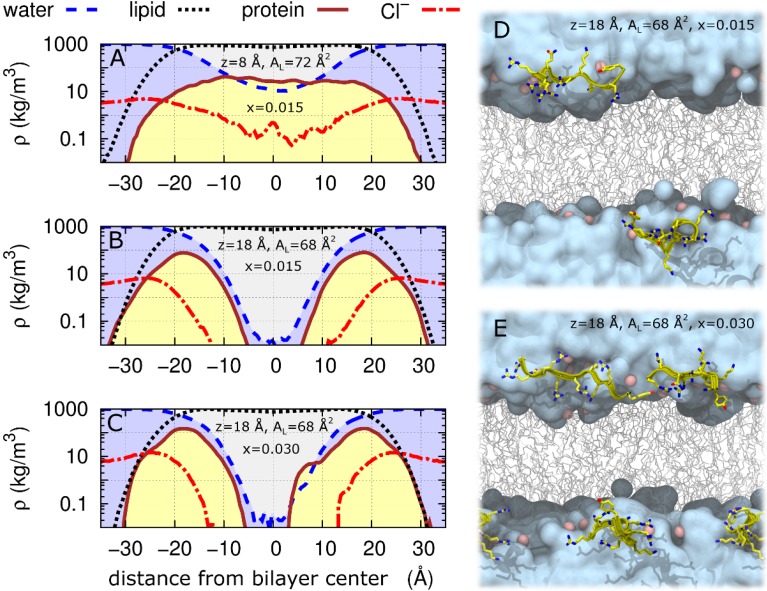
Log scale of mass densities along the DOPC:DOPE (1:1) bilayer normal and snapshots of peptide insertion into the bilayer headgroup region. (**A**–**C**) Average mass density, ρ, of water (dashed blue line), lipid (dotted black line), protein (solid brown line), and Cl^−^ (dash-dot red line) as a function of distance from bilayer center (Å). Simulations restricted to χ^2^ < 3.4 are shown: (**A**) averages of the 10 (of N = 52) simulations at z = 8 Å, A_L_ = 72 Å^2^ and a Tat_47–57_ mole fraction of 0.015; (**B**) all N = 52 simulations at z = 18 Å, A_L_ = 68 Å^2^ and a Tat_47–57_ mole fraction of 0.015; and (**C**) both N = 2 simulations at z = 18 Å, A_L_ = 68 Å^2^ and a Tat_47–57_ mole fraction of 0.030. Snapshots after 100 ns of simulation at z = 18 Å, A_L_ = 68 Å^2^ and a Tat_47–57_ mole fraction of (**D**) 0.015 or (**E**) 0.030. Water is cyan, lipids are gray, lipid headgroup phosphorus atoms are pink, and protein is yellow. Protein is rendered in front of all other molecules for clarity.

The largest value of χ^2^ obtained at z = 18 Å, A_L_ = 68 Å^2^ is 3.4 for Tat_47–57_ mole fractions of either 0.015 or 0.030 (Table 2). Therefore, we define χ^2^ = 3.4 as a cutoff below which simulations are taken to fit the experimental data sufficiently well. Of the N = 52 simulations at z = 8 Å, A_L_ = 72 Å^2^ and a Tat_47–57_ mole fraction of 0.015, there are N = 10 for which χ^2^ ≤ 3.4. Mass density profiles for each molecular type along the bilayer normal averaged over these 10 simulations are shown in Figure 8A. In these simulations, protein molecules often span the bilayer, interacting with the headgroups of both bilayer leaflets and drawing both water and ions into the bilayer’s hydrophobic core (Figure 5B, Figure 7B and Figure 8A).

Average mass density profiles are shown for all N = 52 and N = 2 simulations at z = 18 Å, A_L_ = 68 Å^2^ for Tat_47–57_ mole fractions of 0.015 and 0.030 in Figure 8B,C, respectively. Snapshots of these simulations with the lowest χ^2^ values are depicted in Figure 8D,E.

**Figure 9:**
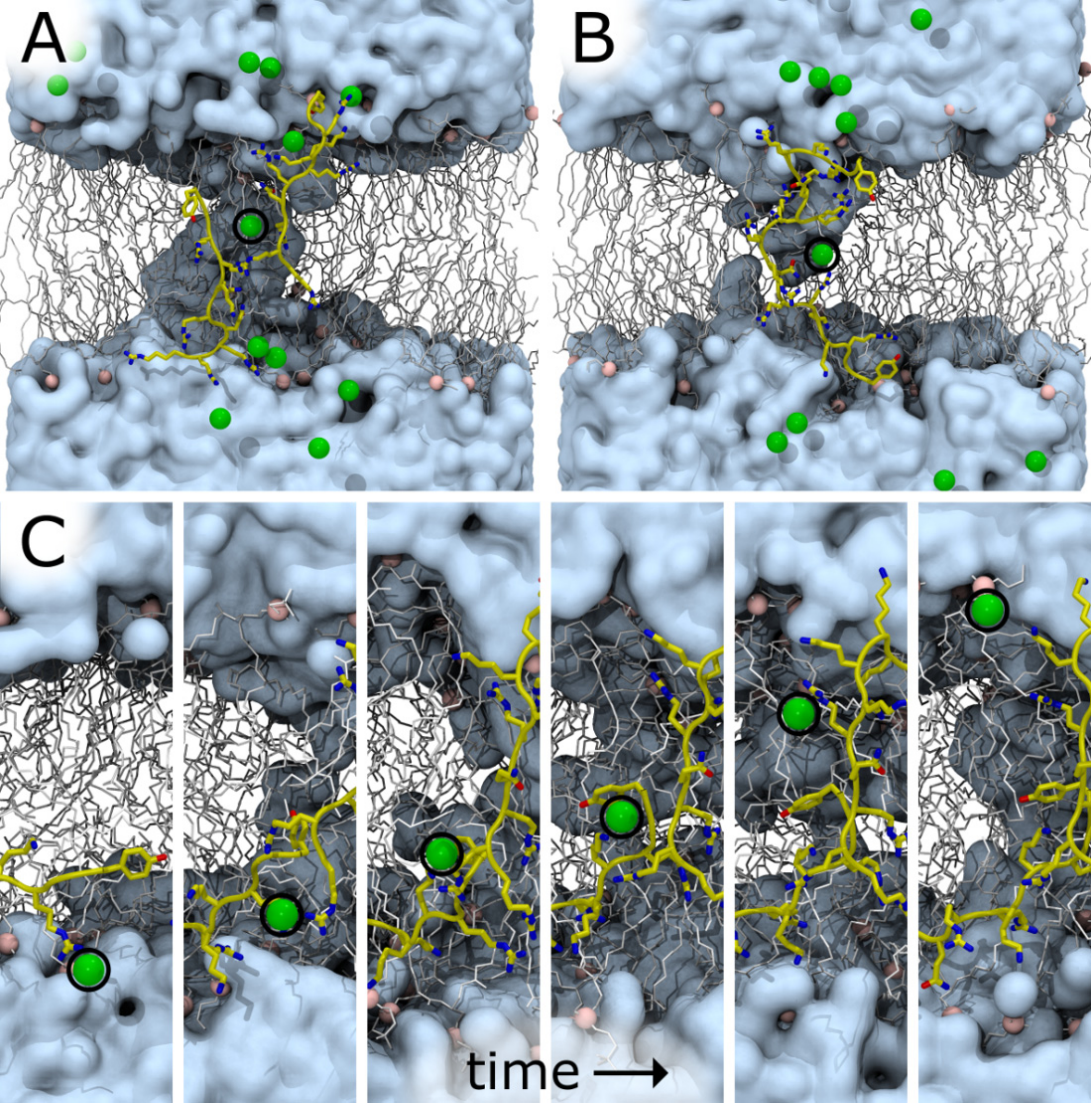
Chloride ion occupancy in the interior of a DOPC:DOPE (1:1) bilayer in simulations at z = 8 Å, A_L_ = 72 Å^2^, x = 0.015. (**A**,**B**) Snapshots at 100 ns in two representative simulations with different initial conformations. χ^2^ values for these simulations are 3.1 (**A**) and 2.8 (**B**). (**C**) Sequential snapshots along a simulation time trajectory in which a Cl^−^ ion crossed the bilayer (χ^2^ = 3.3). Water is cyan, lipids are gray, lipid headgroup phosphorus atoms are pink, protein is yellow, and Cl^−^ is green. Protein and ions are rendered in front of all other molecules for clarity. A black circle is drawn around some ions.

One or more Cl^−^ ions entered the bilayer core in seven of the ten DOPC:DOPE (1:1) simulations at z = 8 Å, A_L_ = 72 Å^2^, x = 0.015 in which χ^2^ < 3.4 (ion distance from the bilayer center along its normal <5 Å). Representative snapshots from two such simulations are shown in Figure 9A,B. In one simulation, a Cl^−^ traversed the bilayer by moving through the column of water surrounding two interacting Tat_47–57_ peptides (Figure 9C).

**Figure 10:**
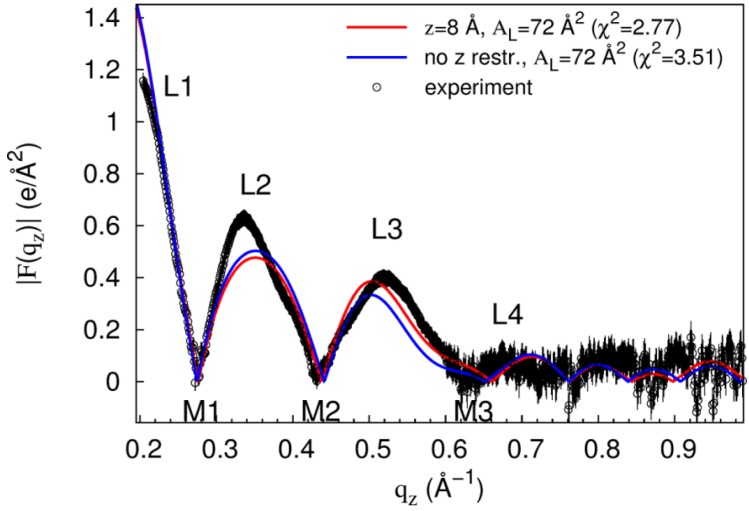
Changes in |F(q_z_)| upon release of peptide restraint. Black circles and error bars are experimental data for DOPC:DOPE (1:1) with a Tat_47–57_ mole fraction of 0.015. Red line is |F(q_z_)| from the simulation at z = 8 Å^2^, A_L_ = 72 Å^2^, x = 0.015 with the smallest χ^2^ value. Blue line is |F(q_z_)| after releasing the restraint on peptide insertion depth. Experimental data were scaled to the |F(q_z_)| from the aforementioned restrained simulation.

To evaluate the conformational stability of the states sampled in restrained simulations, we took the final snapshot from the simulation at z = 8 Å, A_L_ = 72 Å^2^, x = 0.015 that best fits the experimental data (Figure 7B; χ^2^ = 2.8), released the restraints, and simulated for another 100 ns. In this simulation, both peptides retain their interactions with lipid headgroups from both leaflets (not shown), the average values of |z| for the two peptides over the last 50 ns are 6.9 and 9.7 Å, and the form factor computed from the final 50 ns has a χ^2^ value of 3.5 when compared to experiment (Figure 10), indicating that some conformations of Tat_47–57_ insertion into the bilayer core are compatible with unrestrained simulation on the 100-ns timescale. However, substantially longer timescales are required to quantify the stability of such states of deep peptide insertion relative to peptide disposition at the bilayer surface.

At the higher concentration of x = 0.030 (four peptides/128 lipids), our results suggest that Tat_47–57_ resides in the bilayer’s headgroup region and not its hydrocarbon interior. However, in order to make the simulations computationally tractable, our methodological approach relied on the assumptions that all peptides concurrently adopt the same insertion depth and that the local concentration of Tat_47–57_ is homogeneous across spatial scales that are substantially larger than the simulation box (~70 Å). It is therefore possible that at x = 0.030 some subset of the Tat_47–57_ peptide molecules migrate toward the bilayer center. It is also possible that Tat_47–57_ aggregates at x = 0.030, which could affect the peptide’s ability to translocate across a bilayer. However, intermolecular peptide contacts are absent and rare at x = 0.015 and x = 0.030, respectively, in our simulations at z = 18 Å^2^, A_L_ = 68 Å^2^ (Figure S4). Nevertheless, our simulations are much too short to properly assess Tat_47–57_ aggregation (Movie SM2), and further studies are necessary.

## 3. Discussion

Many simulations have attempted to assess the cost of placing either an arginine or an arginine-rich peptide into the membrane hydrocarbon core, which must overcome the energetic barrier of Born self-energy of a charged particle [23]. Table 3 summarizes a representative selection of the published, calculated costs of placing an arginine, or peptides containing arginine, at the center of a lipid bilayer membrane.

In contrast to the high cost of placing an arginine in the center of the bilayer, it has been suggested that the free energy cost for charged amino acids entering the headgroup region is similar to that for partitioning into octanol, about an order of magnitude smaller free energy cost than partitioning into cyclohexane [24]. Similarly, MD simulations suggest that the free energy is smaller for an arginine residing in the interfacial region than in water, by ~3 kcal/mole, depending on the lipid [25]. Specifically, when comparing POPE, whose headgroup occupies a relatively smaller area [26], with POPC, Lindahl *et al.* found a larger free energy for insertion of arginine into the center of a POPE bilayer (26 kcal/mol), compared to a POPC bilayer (19 kcal/mol) [25]. This could have arisen from stronger hydrogen-bonding in the PE headgroup, or because POPE has a thicker hydrocarbon region than POPC [27]. Yet, as stated in the Introduction, the PE headgroup enhances translocation of arginine peptides across membranes, presumably due to its intrinsic negative curvature [11,12]. As measured in the previous Tat study, the bilayer thickness of DOPC:DOPE (1:1) is ~ 2 Å thicker than the corresponding DOPC bilayer [13], yet our current results suggest that at low concentration, Tat_47–57_ may partition closer to the DOPC:DOPE (1:1) bilayer center than in the pure DOPC bilayer. Inclusion of PE may therefore even overcome the effect of an increased thickness. So in spite of the high cost of placing a protonated arginine, or even a Tat peptide at the center of the bilayer, our present result that an interior location of Tat_47–57_ at low concentration in DOPC:DOPE (1:1) membranes may be nearly as good as in the headgroup region, suggests that the curvature-inducing PE headgroup may play an important role.

**Table 3:** Cost of placing charged residues at the bilayer center ^a^.

Charged Species	Simulation/Lipid Membrane	Energetics of Insertion (kcal/mol) [Ref.]
Guanidinium ion	NAMD 2.6/DPPC and CHARMM (continuum)	23 [28]
Protonated Arg on a polyleucine helix	CHARMM27/DPPC	~17 [29,30,31]
Protonated Arg	Gromacs/ DMPC^b^, DOPC, DOTAP^b^, POPC^b^, POPE^b^, POPG^b^	12–26 [25] (lipid dependent)
Protonated Arg	Gromacs 3.3.1/DOPC	~14 [32]
Protonated DiArg ^c^		−1.6 [32]
Protonated TriArg ^c^		−1.1 [32]
Tat_48–60_	Gromacs 3.3.1/DPPC	~32 [33]
Cyclic Arg9—no water pore	Gromacs/DOPC	48 [34]
Cyclic Arg9—in water pore		28 [34]

Notes: ^a^ All simulations are atomistic, unless otherwise defined; ^b^ DMPC, dimyristoylphosphatidylcholine; DOTAP, dioctadecenoyltrimethylammoniumpropane; POPC, palmitoyloleoylphosphatidylcholine; POPE, palmitoyloleoylphosphatidylethanolamine; POPG, palmitoyloleoylphosphatidylcholine; ^c^ Attached to first arginine, which is positioned at the bilayer center.

Also shown in Table 3 is the much lower cost for inserting cyclic Arg into a water pore compared to into the hydrocarbon interior [34]. Our simulations show that when Tat_47–57_ is restrained at 8 Å from the DOPC:DOPE (1:1) bilayer center, water immediately fills the space adjacent to the peptide (Figure 7B and Movie SM1). This result confirms an MD simulation that calculated favorable energetics for water to enter into the hydrocarbon region to stabilize a charged residue [30]. Recall that the hydrocarbon thickness, 2D_C_, decreased by 2.5 Å when Tat_47–57_ is restrained at 8 Å from the DOPC:DOPE (1:1) bilayer center (Table 1). Since A_L_ = Volume_Lipid_ (V_L_)/D_C_ [26], this indicates that V_L_ with Tat_47–57_ and water within the membrane is nearly identical to the neat lipid bilayer, thus leading to a 6 Å^2^ increase in A_L_.

At the higher concentration, Tat_47–57_ is located close to the carbonyl-glycerol group in both DOPC:DOPE (1:1) and DOPC bilayers [13]. This result confirms the results of two previous studies, neutron diffraction of Tat_47–57_ in DOPC:DOPS (80:20) bilayers [35], and NMR of Tat_48–60_ in DMPC:DMPG (8:7) bilayers [36]. A position at the membrane interface allows Tat to quickly translocate across the bilayer if favorable conditions occur.

In the present work, we show that relatively long conformational autocorrelations can exist at values of z near zero, where final conformations and form factors from 100-ns simulations depend strongly on initial conformations (Figure 7 and Table 2). Because the previous study of Tat_47–57_ in DOPC bilayers [13] did not repeat simulations with values of z near the bilayer center using different initial conformations, we are presently unable to quantify the statistical significance of the previous claim made by some of us that Tat_47–57_ does not partition near the center of DOPC bilayers.

The peptide-induced ion transport shown in Figure 9C is consistent with experimental data from Herce *et al.*, which showed that arginine nonamers permeabilize DOPC and DOPC:DOPG (3:1) bilayers to ions [37]. In addition, our simulation results indicate that Tat_47–57_-dependent ionic conduction does not require large toroidal pore structures. This is consistent with experiments in GUVs that showed AlexaFluor647 dye molecules were completely released from PC:PE:cholesterol (50:30:20) vesicles, but that 3 and 70 kDa fluorescent dextrans were not released [12].

Multiple approaches exist to synergistically combine experimental diffraction data with molecular simulation. In one approach, the experimental data is used as a restraint to guide conformational sampling in simulations [38]. In the present work, we adopt an alternative approach in which we rely on experiments to rank-order a set of simulations that are conducted without direct influence from the experimental scattering data. Both approaches have merit and we view them as complementary. In principle, the restrained-simulation approach that we have adopted in this study allows for the computation of free energy profiles or potentials of mean force (PMFs) for Tat_47–57_ peptides along the bilayer normal. However, we have not computed PMFs for two reasons. First, simulations of 100-ns per umbrella are very likely to be subject to systematic sampling errors [15]. Such sampling errors are not necessarily problematic when using simulations to generate models for validation against experimental data, as we do here. However, PMFs require converged sampling and we have therefore chosen not to present this type of data, which we strongly believe would be inaccurate. Second, our sampling along the order parameter of peptide displacement from the bilayer center is discontinuous due to our choice of force constants and z values, and we would need to add new simulations near the bilayer center to construct PMFs.

## 4. Materials and Methods

### 4.1. Experimental Section

#### 4.1.1. Lipids and Peptides

Lipids were purchased from Avanti Polar Lipids (Alabaster, AL, USA) and used without further purification. Dioleoylphosphatidylcholine:dioleolphosphatidylethanolamine (DOPC:DOPE) (1:1) membrane mimics were prepared by first dissolving lyophilized DOPC or DOPE in chloroform and then mixing these stock solutions in a 1:1 mole ratio. Tat peptide (Y_47_GRKKRRQRRR_57_) was purchased from the Peptide Synthesis Facility (University of Pittsburgh, Pittsburgh, PA, USA); mass spectroscopy revealed >95% purity. Tat_47–57_ was dissolved in HPLC trifluoroethanol (TFE) (Sigma, St. Louis, MO, USA) and added to the lipid mixture in mole fractions, x = (Tat_47–57_/lipid + Tat_47–57_), of 0.0087, 0.016, 0.034 and 0.059. The mass of Tat in these mole fractions was corrected for protein content (the remainder being 8 trifluoroacetate counter-ions from the peptide synthesis). Solvents were removed by evaporation in the fume hood.

#### 4.1.2. Samples for X-ray Scattering

Four mg dried lipid/peptide mixture was dissolved in 200 μL HPLC chloroform/TFE (2:1 v:v) and plated onto silicon wafers (15 × 30 × 1 mm^3^) via the rock and roll method [39] to produce stacks of well-aligned bilayers. Solvents were removed by evaporation in the fume hood, followed by 2 h under vacuum.

#### 4.1.3. X-ray Scattering Methods

*LAXS.* Low-angle X-ray scattering data from oriented fluid phase lipid mixtures at 37 °C were obtained at the Cornell High Energy Synchrotron Source (CHESS) using previously described methods [40,41]. Background scattering from the chamber and from water were carefully subtracted as shown in Figure S5. The analysis of diffuse LAXS from oriented stacks of fluctuating fluid bilayers has been previously described [42]. Absolute form factors |F(q_z_)| were obtained as previously described [40]. Further details about data analysis can be found in Supplementary Materials (SM).

### 4.2. Theoretical Section

#### 4.2.1. Simulation Parameters

Molecular dynamics simulations are conducted with a single-precision compilation of version 4.6.5 of the GROMACS simulation package [43]. Protein is modeled by the AMBER99SB-ILDN protein force field [44]. Lipid is modeled by the Stockholm lipid (S-LIPID) parameters [19]. The water model is TIP3P [45]. Bond lengths in protein and lipid are constrained with P-LINCS [46] using sixth-order coupling and a single iteration. Water molecules are rigidified with SETTLE [47]. Lennard-Jones interactions are evaluated using a group-based twin-range cutoff [48] calculated every step for separation distances less than 1.0 nm and every ten steps for distances between 1.0 and 1.4 nm (truncating interactions at 1.4 nm without a smoothing function). Coulomb interactions are calculated using the smooth particle-mesh Ewald (PME) method [49,50] with a Fourier grid spacing of 0.12 nm. Dispersion corrections are applied to both potential energy and pressure [51]. The integration time step is 2 fs. The nonbonded pairlist is updated every 20 fs. Production simulation in the *NpAT* ensemble is achieved as follows (with deviations from this protocol during system setup and equilibration outlined where appropriate). Temperature is controlled using velocity Langevin dynamics [52] at 310 K with a coupling constant of 1 ps. Pressure is coupled semi-isotropically to Parrinello-Rahman barostats [53,54] at 1 bar with coupling constants of 5 ps. Barostat compressibilities are 4.5 × 10^−5^ and 0 bar^−1^ in the Cartesian **z** dimension and **xy** plane, respectively, thus prohibiting changes in the dimensions of the bilayer's transverse plane while allowing fluctuations of the box dimension along the global bilayer normal (the Cartesian **z** axis).

#### 4.2.2. Composite Peptide-Bilayer Systems

Details of peptide and bilayer construction and equilibration are outlined in the SM text. DOPC:DOPE(1:1) simulation systems consist of a hydrated 128-lipid bilayer with 32 DOPC and 32 DOPE lipids and either one or two Tat_47–57_ peptides in each leaflet (total of two or four peptides per system). There are a total of 128 sets of simulation conditions for the pairwise combination of A_L_ values of 62, 64, 66, 68, 70, 72, 74, or 76 Å^2^, 2 or 4 peptides, and displacements between the peptide and bilayer centers of mass along the bilayer normal, z, of 0, 5, 8, 10, 12, 14, 16, or 18 Å, where 0 Å indicates the bilayer center (a given simulation uses the same value of z separately for each peptide). Two simulations are conducted for each of the aforementioned conditions using different initial conformations. Each simulation system is constructed independently using bilayer and peptide conformations drawn from the bilayer- and peptide-only simulations outlined in the SM text. To construct a unique bilayer environment for each simulation, we randomly select a snapshot from the last 10 ns of our 20-ns simulation of a neat bilayer at the desired A_L_, translate it by a random distance in the bilayer plane (resetting the periodic unit cell), and randomly decide whether to rotate the bilayer by 180° to exchange upper and lower leaflets. For each peptide that is to be inserted, we randomly select a snapshot from the last 30 ns of our 40-ns simulation of Tat_47–57_ in water and rotate it with randomly selected magnitudes about each of the three Cartesian axes. Peptides are arranged in the bilayer plane so as to maximize the separation of their centers of mass. Specifically, for simulation systems with two Tat_47–57_ molecules, one peptide is placed at the desired value of z in the +x, +y quadrant of the upper bilayer leaflet and the other peptide is placed in the −x, −y quadrant of the lower leaflet (Figure S6A). Analogously, simulation systems with four Tat_47–57_ molecules have peptides initially placed in the +x, +y and −x, −y quadrants of the upper leaflet and the −x, +y and +x, −y quadrants of the lower leaflet (Figure S6B).

The actual embedding of the peptide in the bilayer is conducted with 40 cycles of the InflateGRO2 routine [55] and double-precision GROMACS steepest descent energy minimization. During this procedure, no lipids are removed from the bilayer. Each system is re-hydrated while disallowing water insertion into the bilayer’s hydrophobic core (7534 +/− 77 water molecules per simulation system), neutralized with chloride ions, and energy minimized. There is no excess salt. The GROMACS pull-code is used to harmonically restrain the center of mass of each peptide to the desired value of z with a force constant of 3000 kJ/mol. This force constant permits an average displacement from the restrained position of approximately 0.1 nm and maximal displacements on the order of 0.5 nm. Each system is equilibrated for 25 ps using Langevin dynamics with a 0.5 fs integration timestep and no pressure coupling. Subsequently, 1 ns of simulation is conducted with Berendsen barostats. Finally, 100 ns of simulation is conducted with Parrinello-Rahman barostats. In each of these simulations, the first 50 ns is discarded as equilibration and the final 50 ns is used for analysis. This timescale is sufficient to relax some conformational degrees of freedom, including local bilayer distortion and water penetration into the bilayer core given the peptide’s conformation, much of which occurs in the first 10 ns (e.g., Movie SM1). However, previous work indicates that structural convergence occurs on the 10 μs timescale [15], and large-scale transitions between locally stable states of peptide-bilayer interaction are unlikely to occur in our 100-ns simulations. In this work, rather than attempt to obtain converged sampling, we rely heavily on experimental data to discriminate against simulations whose form factors are inaccurate either due to the values of z and A_L_, or because they are trapped in the wrong metastable state.

Simulations with pure DOPC bilayers are set up and conducted analogously for *A*_L_ values of 66, 68, 70, 72, 74, or 76 Å^2^; here, each condition is simulated only once.

#### 4.2.3. Comparing Form Factors from Experiment and Simulation Data

Electron density profiles along the Cartesian **z** axis are constructed from the last 50 ns of each simulation trajectory using a modified version of the g density tool from GROMACS 5.0.4 (this custom modification ensures that the bilayer is properly centered in each trajectory frame). These density profiles are converted through the Fourier transform to form factors, |F(q_z_)|, using the SIMtoEXP software [56].

Values of |F(q_z_)| evaluated from simulation are well defined and are expressed in units of e/Å^2^. However, the experimental values of |F(q_z_)| include an unknown scaling factor. Therefore, when evaluating the goodness of fit between simulation and experimental data, one may (and should) scale either form factor to minimize the inter-form factor deviation. To facilitate comparison of measures of the goodness of fit between experimental data sets, we initially scale all experimental form factors such that the maximum value in the second lobe is equal to one (e.g., Figure S7A). We subsequently set the experimental error, ∆, equal to 0.05 and 0.1 e/Å^2^ for q_z_ < 0.6 and q_z_ ≥ 0.6 Å^−1^, respectively.

The simulation scaling factor, a^sim^ is selected separately for each simulation according to:

(1) $$a^{\text{sim}}=\frac{\sum_{i=1}^{N}{\left(\frac{1}{\Delta_{i}^{\exp}}\right)}^{2}\left|F_{i}^{\exp}\right|\left|F_{i}^{\text{sim}}\right|}{\sum_{i=1}^{N}{\left(\frac{1}{\Delta_{i}^{\exp}}\right)}^{2}{\left|F_{i}^{\text{sim}}\right|}^{2}}$$

for all N data points after linearly interpolating the values of |F(q_z_)| from simulation such that q_z(i)_ from simulation and experiment are equal for all data points.

Next, we quantify the goodness of fit between experiment and simulation, χ^2^, as:

(2) $$\chi^{2}=\frac{\sum_{i=1}^{N}{\left(\frac{1}{\Delta_{i}^{\exp}}\times \left[\left|F_{i}^{\exp}\right|-a^{\text{sim}}\times \left|F_{i}^{\text{sim}}\right|\right]\right)}^{2}}{N-1}$$

As outlined above, we scale the values of |F(q_z_)| from simulation to match those from experiment in the determination of χ^2^ (Equations (1) and (2)). All plots of |F(q_z_)| comparing results from simulation and experiment use unscaled ordinate values (expressed in units of e/Å^2^) from the simulation having the lowest χ^2^ value; experimental values of |F(q_z_)| (already scaled such that the maximum value in lobe 2 equals one) are multiplied by 1/a^sim(best)^, where a^sim(best)^ is the value of a^sim^ obtained for the simulation having the lowest χ^2^ value; and all other simulation values of |F(q_z_)| are multiplied by a^sim^/a^sim(best)^, where a^sim^ differs for each simulation.

All reported values of χ^2^ are taken straight from Equations (1) and (2) and are reported without further modification. Additional discussion of fitting procedures is included in the SM text.

## 5. Conclusions

We use atomistic molecular dynamics simulations to identify bilayer insertion depths of the Tat_47–57_ peptide from HIV-1 that are maximally consistent with experimental DOPC:DOPE (1:1) bilayer form factors derived from X-ray diffuse scattering data. Whereas Tat_47–57_ appears to reside exclusively at the surface of DOPC bilayers [13], we show that the penetration of this peptide into the bilayer’s hydrophobic core may be stabilized by the addition of PE lipids. Slow conformational relaxation of the peptide and/or bilayer on the 100 ns simulation timescale prevents us from drawing mechanistic conclusions about the nature of PE-assisted Tat_47–57_ bilayer penetration. Nevertheless, our results suggest that Tat_47–57_ translocation across PE-rich bilayers may involve a metastable intermediate caused by the tendency for PE to adopt a negative curvature, in which monomeric and/or dimeric peptides adopt an extended conformation and interact with the headgroups of lipids from both bilayer leaflets, drawing both water and chloride ions into the bilayer interior.

## Supplementary Materials

Additional Methods, seven Figures, and References [13,57,58,59,60,61,62,63] are presented in the Supplementary Materials.
