# Supplementary material for: Penetration of HIV-1 Tat47–57 into PC/PE Bilayers Assessed by MD Simulation and X-ray Scattering
Source: Membranes (Basel). 2015 Sep 22;5(3):473–94. doi: 10.3390/membranes5030473 (PMC4584291; doi:10.3390/membranes5030473)
Supplement: Supplementary file 1 [file membranes-05-00473-s001.zip › membranes-99006-suppl-final/membranes-99006-suppl-final.pdf]

Supplementary Materials

1. Supplemental Method

1.1. MD Simulation

Bilayer preparation: A configuration of a DOPC:DOPE (1:1) lipid bilayer with 64 lipids per leaflet is obtained from the CHARMM-GUI [1] membrane builder [2] (with 32 DOPC and 32 DOPE lipids in each leaflet). This bilayer conformation is solvated with 8100 water molecules, energy minimized, and simulated for 100 ns using stochastic velocity rescaling [3] for temperature control with a coupling constant of 1 ps, employing separate temperature coupling groups for lipid and water. Pressure coupling is via semi-isotropic Berendsen barostats [4] with coupling constants of 1 ps and compressibilities of 4.5×10^{-5} bar^{-1} in each of the Cartesian **z** dimension and **xy** plane. In the last 50 ns of this trajectory, the average area per lipid (A_L) is 62 Å^2 (σ = 1 Å^2; throughout this article, σ denotes the standard deviation of the sample, whereas ± denotes the standard error of the mean). The final snapshot from this 100-ns trajectory is used to create systems with A_L values of 62, 64, 66, 68, 70, 72, 74, and 76 Å^2 by enlarging the simulation box in the global bilayer plane. Each of these systems is simulated for 20 ns to generate trajectories from which to draw initial conformations for simulations with $Tat_{47\text{-}57}$. The final snapshot from each of these 20-ns simulations is used to initiate a new 100 ns simulation at the specified value of A_L using the production simulation conditions noted in the previous section (*i.e.*, Langevin dynamics with a single temperature coupling group and Parrinello-Rahman barostats). The final 50 ns of these 100-ns simulations of neat bilayers are used for comparison to experiment.

Pure DOPC bilayers are constructed and simulated similarly.

Peptide preparation: An all-*trans* structure of the $Tat_{47–57}$ peptide ($Y_{47}GRKKRRQRRR_{57}$) with zwitterionic backbone termini is prepared with the VMD Molefacture plugin [5]. This structure is placed in a rhombic dodecahedral box (volume = 100 nm^3), hydrated, neutralized with eight Cl^- ions, energy minimized, and simulated for 40 ns. This simulation uses isotropic pressure coupling but otherwise employs the parameters outlined above for bilayer preparation (ions are grouped with water for temperature coupling).

Simulations of neat bilayers with variable A_L: To assess the existence of any potential artifacts in $|F(q_z)|$ resulting from fixing the A_L value, we conduct a 500-ns simulation for each of the neat DOPC and DOPC:DOPE (1:1) bilayers in which the A_L is variable. These simulations are set up and conducted according to the procedure listed in the main text for neat bilayer simulations at fixed A_L, except that barostat compressibilities are 4.5×10^{-5} in both the Cartesian **z** dimension and **xy** plane. $|F(q_z)|$ are based on the last 450 ns of each simulation after discarding the first 50 ns as equilibration. The average A_L values sampled by these simulations are 67.6 ± 0.37 Å^2 (σ = 1.3 Å^2) and 63.7 ± 0.37 Å^2 (σ = 1.2 Å^2) for simulations of DOPC and DOPC:DOPE (1:1) bilayers, respectively. Standard deviations of the mean are obtained by block averaging [6] using 50-ns segments. We draw two important conclusions from these simulations. First, when comparing variable-A_L simulations to simulations with A_L fixed near the average A_L sampled in the variable-A_L simulations, $|F(q_z)|$ are similar (Figure S1). Therefore, even though it is possible that fixing the A_L perturbs $|F(q_z)|$ when fluctuations in A_L would otherwise be very large, we do not observe such an effect. Second, some of our simulations at fixed A_L have $|F(q_z)|$ that fit the experimental $|F(q_z)|$ better than the simulations with variable A_L (Figure S1).

This inability of the variable-A_L simulations to maximally fit the experimental $|F(q_z)|$ provides further support for the systematic approach that we have adopted in this work, in which we rely on experiment to discriminate against poor simulation models rather than demanding that simulation alone accurately reproduces the biomolecular systems under investigation.

Comparing form factors from experiment and simulation: Our choice to scale simulation to match experiment as outlined in Equation (1) (rather than the inverse scaling of experiment to match simulation) makes one set of $|F(q_z)|$ values constant across all fits, thus avoiding problems related to variation in the average magnitude of $|F^{sim}(q_z)|$. Specifically, scaling the experimental data to fit the simulation data using an analytical equation to determine a^{exp} cannot account for the contribution to χ^2 due to the change in the estimated experimental error, $\Delta(q_z)$, (which in that case should be scaled by a^{exp}) and analytical determination of a^{exp} is possible only when all $\Delta(q_z) = 1$ (in Equations (1) and (2), q_z is linearized and $\Delta(q_z)$ is represented by Δ_i). The two obvious solutions are to (a) use a minimization algorithm to select a^{exp} such that it minimizes the value of χ^2, or (b) scale the simulation data to fit the experimental data, which does not require scaling of $\Delta(q_z)$. We have adopted approach (b) in this work.

We choose to define the experimental errors to be 0.05 or 0.1 e/Å^2 rather than using the standard deviations at each value of $|F(q_z)|$ from repeat experiments because there is uncertainty in the estimates of the experimental error and aberrantly small values of Δ_i, which appear in the denominator in Equation (2), overestimate of the importance of some deviations that then dominate the final value of χ^2. The precise values we use for the experimental error (0.05 and 0.1 e/Å^2) are based on visual assessment of the standard deviation of $|F(q_z)|$ among repeat experiments (e.g., Figure S3B).

All reported values of χ^2 are taken from Equations (1) and (2) and are reported without further modification. These values of χ^2 are therefore defined up to a multiplicative constant and can only be rigorously compared within a set of simulations that are scaled to fit a single experimental data set. However, because the experimental profiles of $|F(q_z)|$ are initially scaled such that the maximal value of the second lobe is equal to one (Figure S7A), these χ^2 values can be compared between different bilayer compositions and peptide concentrations. Although rescaling χ^2 values based on the value of $a^{sim(best)}$ for each molecular composition would allow rigorous comparison of the goodness of fit between these different data sets, we have chosen not to do this for two reasons. First, our main aim of identifying simulation models that best fit experimental data only requires comparison of χ^2 values within a group of simulations that are all compared to the same experimental data. Second, rescaling χ^2 values in this way would make the fitness quantification dependent on the results of a few simulations, which may be less reproducible than the experimental data.

1.2. X-ray Experiments

Experimental methods are described in the main paper and also in Ref. [7]. After X-ray diffuse scattering data are collected, backgrounds are carefully subtracted so that they do not interfere with fitting to the liquid crystal theory [8], and to best determine the small intensity lobes at high q_z. Figure S1 shows the method of signal processing of diffuse X-ray scattering images.

Once the backgrounds are carefully subtracted as shown in Figure S5, the remaining scattered intensity (white lobes in Figure S1) is used to obtain the experimental form factors, using the equation: $I(\mathbf{q}) = S(\mathbf{q})\ |F(q_z)|^2/q_z$, where $\mathbf{q} = (q_z, q_r)$, $I(\mathbf{q})$ = intensity, $S(\mathbf{q})$ is the structure factor, $|F(q_z)|$ is the

bilayer form factor whose Fourier transform is the electron density profile ρ(z), and q_z^{-1} is the usual Lorentz factor for oriented samples [9]. The resulting $|F(q_z)|$ is used as a model-free comparison to the $|F(q_z)|$ resulting from the MD simulations.

For the DOPC:DOPE (1:1) bilayers, experimental form factors are the averages of 3, 2, and 9 separate experiments for neat bilayers and bilayers with $Tat_{47–57}$ mol fractions of 0.016 and 0.034, respectively.

The intensities of out-of-plane scattering, $I(q_z)$, have a statistical noise component that is roughly Gaussian. Because the absolute value of the form factor, $|F(q_z)|$, depends on the square root of $I(q_z)$, negative values of $I(q_z)$ would result in imaginary values of $|F(q_z)|$. In plots of $|F(q_z)|$, it is convenient to represent these negative values of $I(q_z)$ as negative values of $|F(q_z)|$. Although negative values of $|F(q_z)|$ are unphysical, it is important that they be retained in the fitting procedure [9]. To understand why, consider values of q_z in which, absent noise, $|F(q_z)| = 0$. Retaining only positive values of $|F(q_z)|$ would bias the fit to a positive value when it should be zero. It may also be noted that taking the square root of $I(q_z)$ to obtain $|F(q_z)|$ makes the statistical noise in $|F(q_z)|$ when $|F(q_z)| = 0$ both bimodal and magnified in amplitude compared to the noise when $|F(q_z)| >> 0$.

2. Supplemental Figures

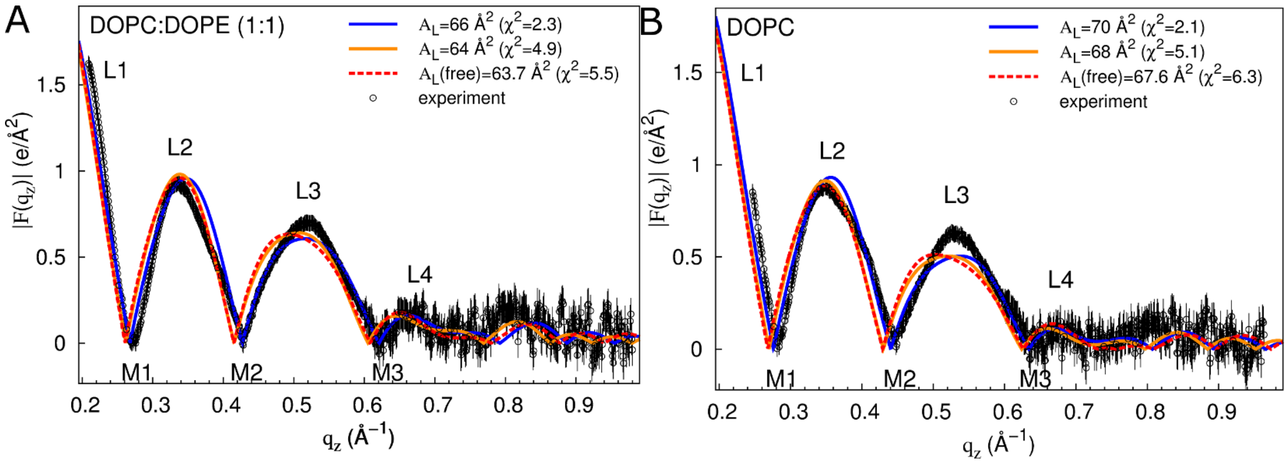

Figure S1. Form factor comparison for neat bilayer simulations with and without fixed A_L. Data show $|F(q_z)|$ for neat bilayers composed of (**A**) DOPC:DOPE (1:1) and (**B**) DOPC. $|F(q_z)|$ from experiment is shown in black circles with vertical lines depicting experimental error estimates (see Methods). $|F(q_z)|$ for the fixed-A_L simulation with the smallest χ^2 value is shown as a blue line. $|F(q_z)|$ for the variable-A_L (free) simulation is shown as a dashed red line. $|F(q_z)|$ for the fixed-A_L simulation whose A_L is closest to the average A_L value sampled in the variable-A_L simulation is shown as an orange line. χ^2 values are in parentheses. Out-of-plane scattering lobes (L) and minima (M) are numbered based on experimental data. Experimental data were scaled to the simulated $|F(q_z)|$ with the smallest χ^2.

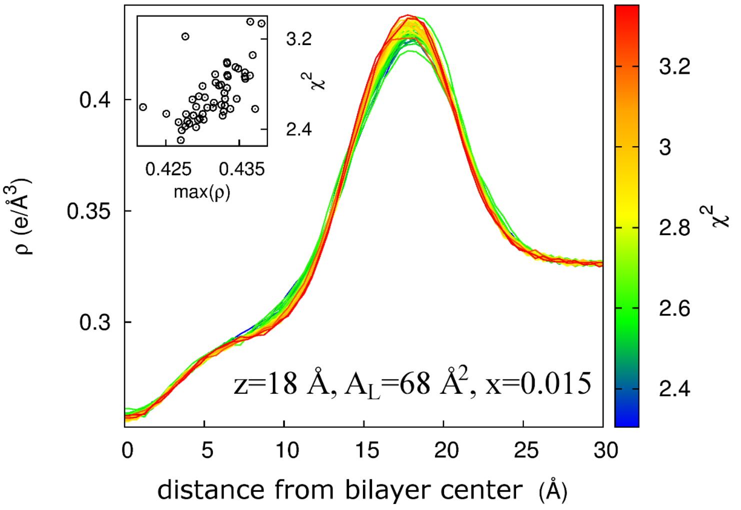

Figure S2. Bilayer undulations do not increase χ^2 at z = 18 Å, A_L = 68 Å^2, x = 0.015. Total system electron density, ρ, as a function of absolute distance from the bilayer center along its normal, for N = 52 independent simulations, colored by χ^2 values representing a range from best (blue) to worst (red) fit to experiment. Inset, χ^2 as a function of the maximum value of ρ.

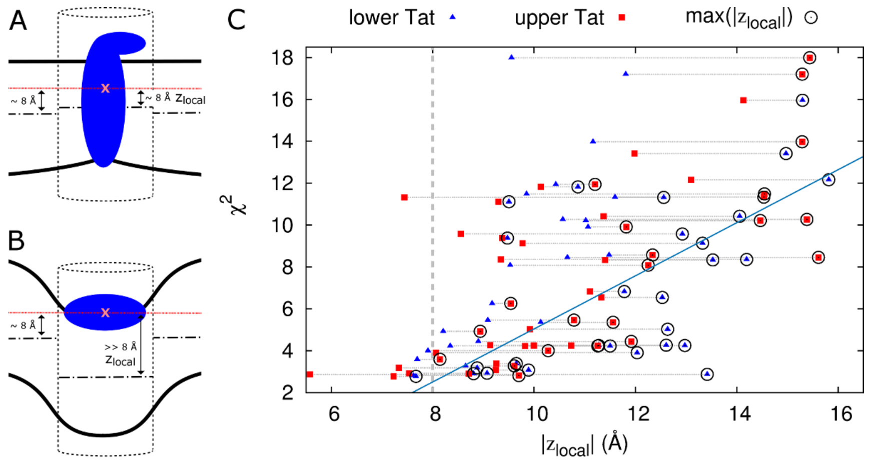

Figure S3. Local peptide insertion depth and correlation with χ^2 at z = 8 Å, A_L = 72 Å^2, x = 0.015. (**A** and **B**) Schematic depictions of the difference between the distance of the peptide's center of mass from the global bilayer center, z, and the local bilayer center, z_{local}. Images represent lipid headgroups (black solid lines), protein (blue shape), protein center of mass (red "x" and dotted line), and global and local centers of mass (black dash-dot lines) of the lipid bilayer. Dashed cylinder contains lipid atoms within a 1 nm cylinder centered around the peptide center of mass and aligned with the global bilayer normal (Cartesian **z** axis), which is used for the definition of z_{local}. (**C**) χ^2 values are shown as a function of the absolute value of z_{local}. Gray vertical dashed line represents z = 8 Å. Blue triangles and red squares show $|z_{local}|$ for lower and upper leaflets, respectively. For each simulation, the maximum value of $|z_{local}|$ is circled. Horizontal lines connect values of $|z_{local}|$ sampled by the two peptides in each simulation. Cyan line shows the linear best fit: $\chi^2 = 1.27 \times \max(|z_{local}|) - 7.63$, for which $r^2 = 0.49$. $|z_{local}|$ correlates on average with χ^2 at z = 8 Å, A_L = 72 Å^2, x = 0.015, but is not predictive.

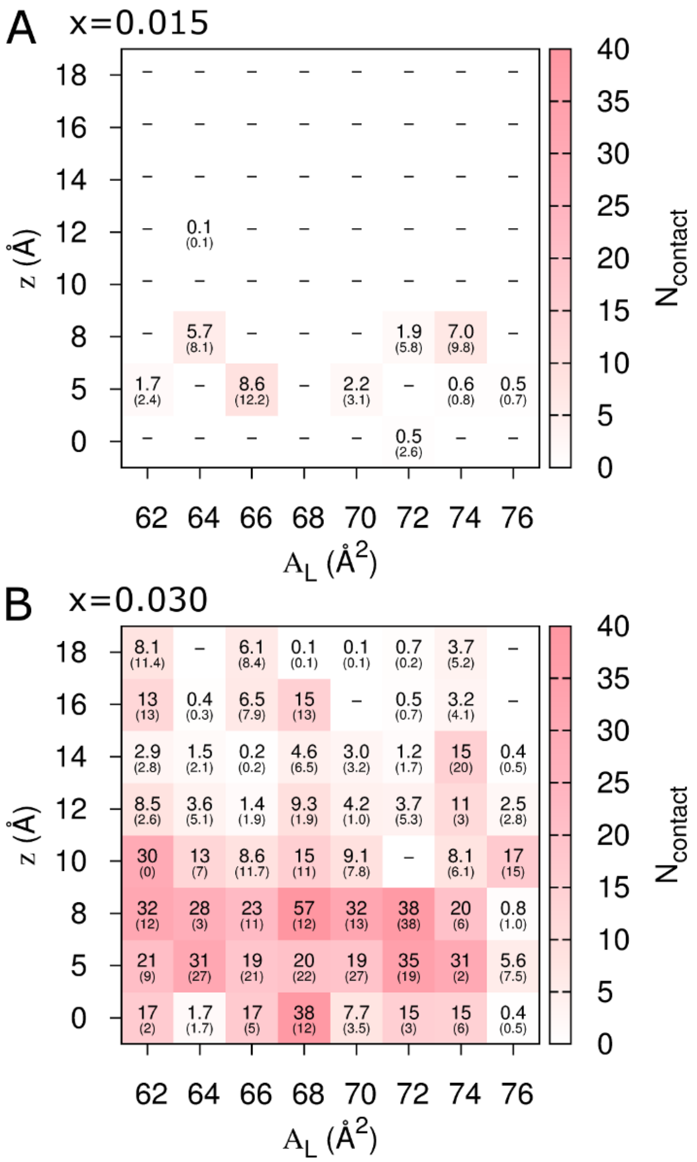

Figure S4. Quantification of aggregation in DOC:DOPE (1:1) simulations with a Tat$_{47–57}$ mole fraction of 0.015 (**A**) and 0.030 (**B**). Color and inset numbers represent N$_{contact}$, the number of intermolecular contacts (within 6 Å) between peptide non-hydrogen atoms (standard deviations of averages from repeat simulations in parentheses). Data at x = 0.015 for z = 18 Å^2, A$_L$ = 68 Å^2; z = 8 Å^2, A$_L$ = 72 Å^2; and z = 0 Å^2, A$_L$ = 72 Å^2 are from all N = 52 simulations.

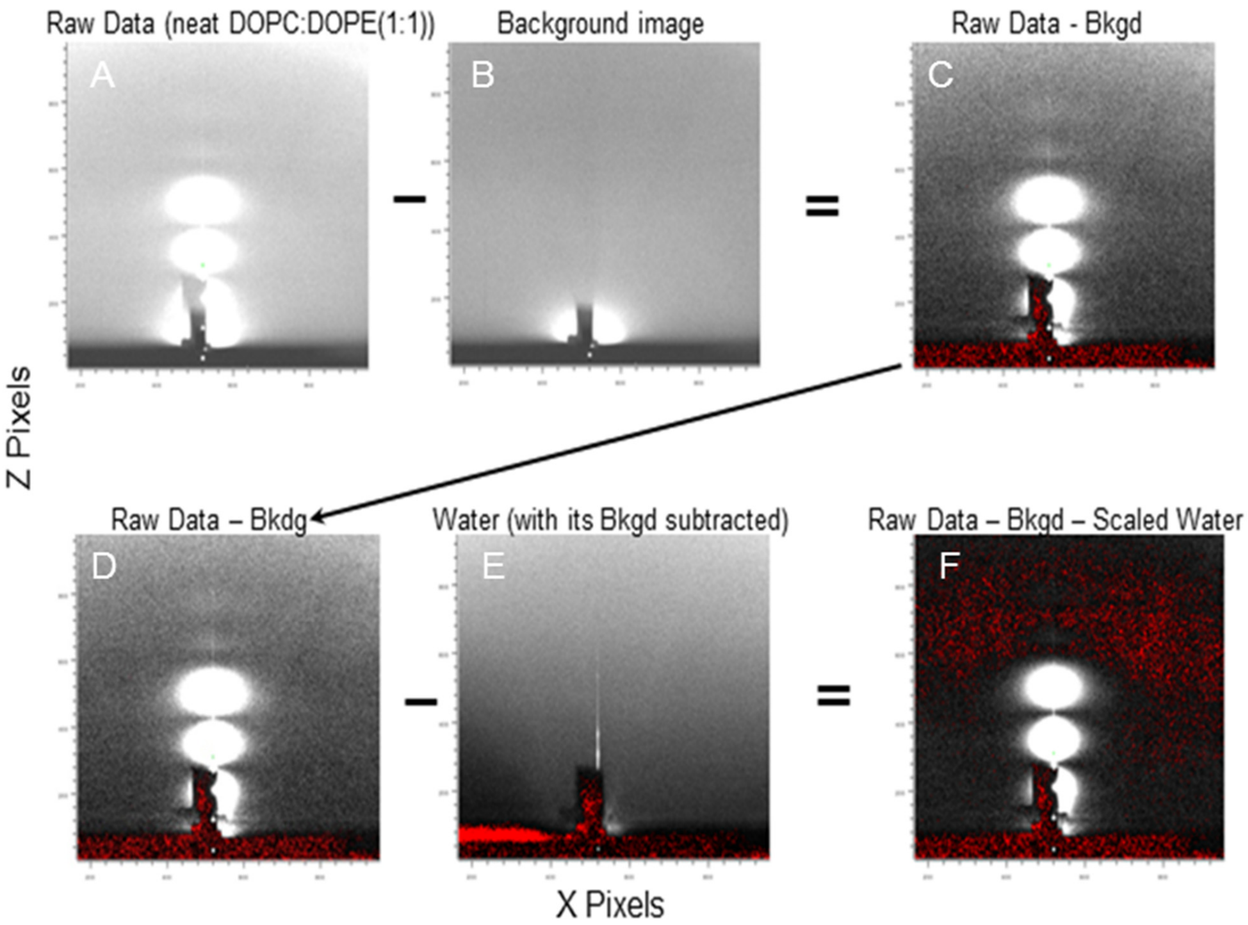

Figure S5. Signal processing of diffuse X-ray scattering CCD images. Image A is the raw data of neat DOPC:DOPE (1:1) bilayers. From this is subtracted a background image (B), which is obtained by exposing the back side of the sample's silicon wafer to X-rays for the same time as the raw data image yielding C. A second background consisting of water condensed onto the silicon wafer (E) is then scaled to match the diffuse background data near the top of the image in D. The final image with these two backgrounds subtracted is shown in F. (**A**) Raw data; (**B**) Background image; (**C**) Raw data - background image; (**D**) Same as C; (**E**) Water background – its background image; (**F**) Raw data – background image – scaled water.

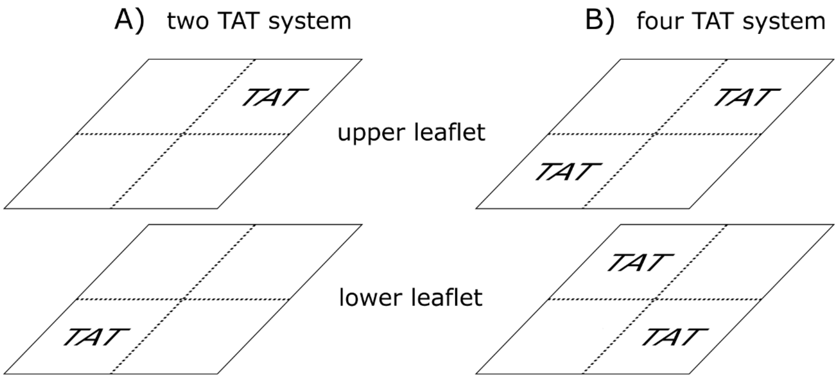

Figure S6. Initial placement of $Tat_{47–57}$ peptides in the bilayer. (**A**) two TAT system; (**B**) four TAT system.

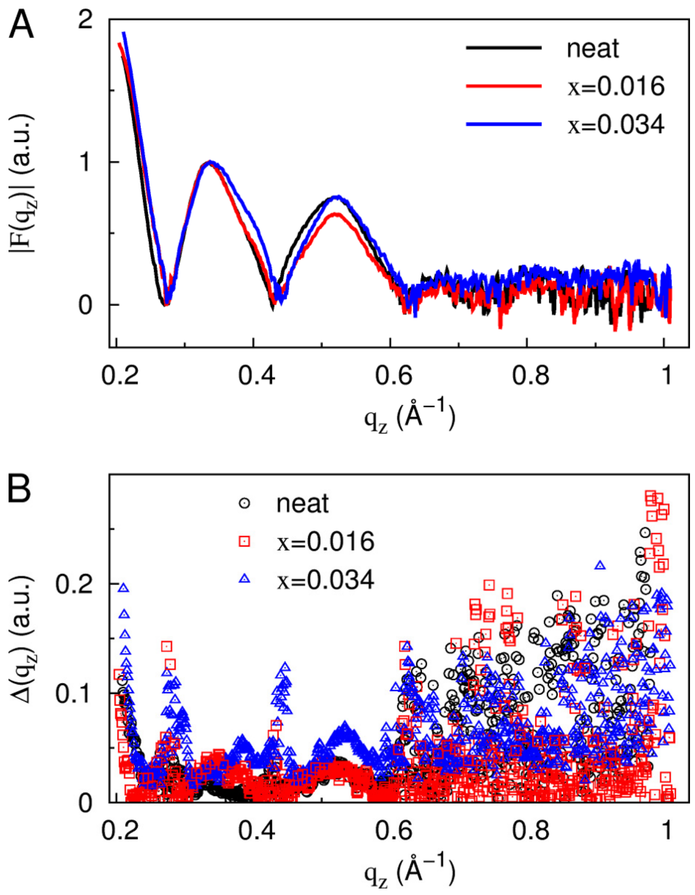

Figure S7. Experimental form factors for a 1:1 DOPC:DOPE bilayer with increasing $Tat_{47–57}$ mole fractions, $x = Tat_{47–57}/(Tat_{47–57} + \text{lipid})$. (**A**) Form factors, $|F(q_z)|$, scaled such that the maximal value in the second lobe is 1.0 arbitrary units (a.u.). (**B**) Estimated experimental error, $\Delta(q_z)$, after scaling, but before resetting the $\Delta(q_z)$ values to account for uncertainty in the estimated experimental error as described in the Supplemental Methods section titled "Comparing form factors from experiment and simulation" and the subsequent text in this caption. During determination of χ^2 values, $\Delta(q_z)$ is set to 0.05 and 0.1 e/Å^2 for $q_z < 0.6$ and $q_z \geq 0.6$ Å^{-1}, respectively. These final values of $\Delta(q_z)$ were selected by eye based on part B in this Figure.

3. Supplemental Movies

3.1. Movie SM1

Representative trajectory of $Tat_{47–57}$ in DOPC:DOPE (1:1) at $z = 8$ Å, $A_L = 72$ Å^2, $x = 0.015$ with a relatively good $|F(q_z)|$ fit to experiment. This simulation has a χ^2 value of 2.9 and is one of the two trajectories averaged together to generate the $|F(q_z)|$ shown as a dark blue line in Figure 3B. Movie shows the entire trajectory, while $|F(q_z)|$ is based on the last 50 ns. Water is cyan, lipids are grey, lipid headgroup phosphorus atoms are pink, protein is yellow, and Cl^- is red. Protein is rendered in front of all other molecules for clarity.

3.2. Movie SM2.

Representative trajectory highlighting the relatively slow diffusion of $Tat_{47–57}$ along the DOPC:DOPE (1:1) bilayer surface (z = 18 Å). Simulations at x = 0.030 have two peptides per bilayer leaflet, but the 100-ns timescale is insufficient to quantify the peptide's propensity to aggregate, which would require many association and dissociation events. Lipids are grey with pink headgroup phosphorus atoms and protein is yellow.

Supplemental References

1. Jo, S.; Kim, T.; Iyer, V.G.; Im, W. Charmm-gui: A web-based graphical user interface for charmm. *J. Comput. Chem.* **2008**, *29*, 1859–1865.
2. Jo, S.; Lim, J.B.; Klauda, J.B.; Im, W. Charmm-gui membrane builder for mixed bilayers and its application to yeast membranes. *Biophys. J.* **2009**, *97*, 50–58.
3. Bussi, G.; Donadio, D.; Parrinello, M. Canonical sampling through velocity rescaling. *J. Chem. Phys.* **2007**, *126*, doi:10.1063/1.2408420.
4. Berendsen, H.J.C.; Postma, J.P.M.; van Gunsteren, W.F.; DiNola, A.; Haak, J.R. Molecular dynamics with coupling to an external bath. *J. Chem. Phys.* **1984**, *81*, 3684–3690.
5. Humphrey, W.; Dalke, A.; Schulten, K. Vmd: Visual molecular dynamics. *J. Mol. Graphics* **1996**, *14*, 33–38.
6. Flyvbjerg, H.; Petersen, H.G. Error estimates on averages of correlated data. *J. Chem. Phys.* **1989**, *91*, 461–466.
7. Akabori, K.; Huang, K.; Treece, B.W.; Jablin, M.S.; Maranville, B.; Woll, A.; Nagle, J.F.; Garcia, A.E.; Tristram-Nagle, S. HIV-1 Tat membrane interactions probed using X-ray and neutron scattering, Cd spectroscopy and MD simulations. *BBA Biomembr.* **2014**, *1838*, 3078–3087.
8. Lyatskaya, Y.; Liu, Y.; Tristram-Nagle, S.; Katsaras, J.; Nagle, J.F. Method for obtaining structure and interactions from oriented lipid bilayers. *Phys. Rev. E Stat. Nonlin Soft Matter Phys.* **2001**, *63*, doi:10.1103/PhysRevE.63.011907.
9. Kucerka, N.; Liu, Y.; Chu, N.; Petrache, H.I.; Tristram-Nagle, S.; Nagle, J.F. Structure of fully hydrated fluid phase dmpc and dlpc lipid bilayers using X-ray scattering from oriented multilamellar arrays and from unilamellar vesicles. *Biophys. J.* **2005**, *88*, 2626–2637.

© 2015 by the authors; licensee MDPI, Basel, Switzerland. This article is an open access article distributed under the terms and conditions of the Creative Commons Attribution license (http://creativecommons.org/licenses/by/4.0/).
